# Inter-evaluator bias and applicability of feline body condition score from visual assessment

**DOI:** 10.3389/fvets.2025.1604557

**Published:** 2025-08-18

**Authors:** Emily C. Graff, Christopher R. Lea, Diane Delmain, Erin D. Chamorro, Xiaolei Ma, Jingyi Zheng, Yue Zhang, Emily Brinker, Kenzii Kittell, Mackenzie Hicks, Casey Pfister, Heather Hamilton, Qinghong Li, Douglas R. Martin, Xu Wang

**Affiliations:** ^1^Department of Pathobiology, College of Veterinary Medicine, Auburn University, Auburn, AL, United States; ^2^Scott-Ritchey Research Center, College of Veterinary Medicine, Auburn University, Auburn, AL, United States; ^3^Department of Clinical Sciences, College of Veterinary Medicine, Auburn University, Auburn, AL, United States; ^4^Department of Mathematics and Statistics, College of Science and Mathematics, Auburn University, Auburn, AL, United States; ^5^Department of Comparative Pathobiology, Cummings School of Veterinary Medicine, Tufts University, North Grafton, MA, United States; ^6^Veterinary Education Center, College of Veterinary Medicine, Auburn University, Auburn, AL, United States; ^7^Nestlé Research, St. Louis, MO, United States; ^8^Department of Anatomy, Physiology, and Pharmacology, College of Veterinary Medicine, Auburn University, Auburn, AL, United States; ^9^Center for Advanced Science, Innovation, and Commerce, Alabama Agricultural Experiment Station, Auburn, AL, United States; ^10^HudsonAlpha Institute for Biotechnology, Huntsville, AL, United States

**Keywords:** domestic cat, body weight, body condition score, palpation, telemedicine, obesity

## Abstract

Body Condition Score (BCS) is an effective tool for assessing body weight and fat mass, as well as diagnosing obesity and abnormal weight loss. A method for visual assessment of BCS in cats would be useful to expand access for feline health and research. The goal of this study is to determine whether BCS can be accurately assessed solely from photographs of cats, and to measure inter-evaluator bias in visually assessed BCS. To do this, a set of online-sourced cat images was administered as a quiz to nine evaluators. Inter-evaluator bias was relatively low (mean ± SE = 0.35 ± 0.03) with ~50% complete agreement. To validate the results, a BCS was clinically assessed during routine wellness exams for 38 cats, enrolled, through palpation by one evaluator and visual assessment by all nine evaluators using photographs collected at the exam. The visual assessment of BCS deviated from the clinically assessed BCS by 0.61 ± 0.04, which was slightly higher than the deviation observed in the online-sourced image set. In both scenarios, the majority voting among all evaluators achieved the highest accuracy, demonstrating its effectiveness in reducing evaluator bias. Inter-evaluator bias caused a 15.5% misclassification between ideal and overweight BCS but 1.8% between ideal and obese, indicating minimal bias in diagnosing feline obesity. The ability to accurately assess BCS through photographic evaluation will enhance remote consultations in telemedicine and support large-scale epidemiological studies. This study has developed a method for evaluating and minimizing inter-evaluator bias in BCS assessments across diverse practitioners and settings, thereby improving consistency and comparability and improving our understanding and application of BCS as a tool for feline health.

## Introduction

The energy balance model of obesity outlines the pathogenesis of excessive food intake and lack of physical exercise that leads to expansion of adipose tissue, resulting in metabolic dysregulation (1). When excess body fat accumulates to the extent that it has adverse effects on health, it is termed obesity, a condition that is common in companion animals (2, 3). Feline obesity is a major epidemic that currently affects at least 45% of client-owned cats (4, 5) and is considered the second most common health problem in domestic cats in developed countries (6). It is linked to many systemic health conditions, including insulin resistance and type 2 diabetes mellitus (T2DM), dyslipidemia, neoplasia, urinary diseases, and respiratory diseases (2), and reduced lifespan (7). In many species BCS correlates with a number of physiologic processes, including glucose homeostasis and systolic blood pressure in companion animals (8), and meat, fiber, and milk quality in production animals (9–11).

BCS assessment is a critical piece of the physical exam in veterinary medicine to evaluate fat mass and health status in a variety of species. It was first described in horses to provide an objective score of physical measurements and body fat distribution that could be standardized across breeds (12). Over the years, it has been adapted to several species, including cattle (13, 14), dogs (15), poultry (9), exotic pets (16), wildlife (17–19), as well as domestic cats (7, 20, 21). For cats, the BCS assessment is based on a combination of visual and physical evaluation and commonly scored on a 9-point scale originally developed in 1997 (22). The scale creates a repeatable and reproducible a semi-quantitative assessment that correlates well with percent body fat as determined by dual-energy X-ray absorptiometry (DEXA) (20, 23). Typically, a score of 1 ~ 3 indicates that the animal is too thin or underweight, scores of 4 ~ 5 represent ideal body condition, 6 ~ 7 are overweight, and 8 ~ 9 are obese (20, 22, 23), though recent evaluation suggests that a BCS of 5–6 on a 9 point scale may be more consistent with ideal (7).

Regardless of the system used, BCS is an essential component of the patient evaluation in feline practice, providing a standardized method for assessing a cat’s body fat and overall health status. In both dogs and cats, BCS systems have been associated with life expectancy or longevity (7, 24), with scores below and above an ideal BCS negatively associated with survival and lifespan in cats (7). One study noted that even a change of one BCS score on the 5-point scale could impact feline life expectancy (24).

The most common use of BCS in companion animals in modern veterinary medicine is as a metric for increased fat mass as obesity is the most common nutritional problem in dogs, cats and many domestic and exotic pets (3) and can vary among different breeds with important impacts on health (21). In the context of obesity research, BCS serves as a fundamental metric for investigating and analyzing the prevalence, risk factors (6, 25), metabolic and microbiome correlates (26–28), molecular mechanisms (29), and consequences of excess body weight in domestic cats. In the original study establishing the 9-point BCS system, BCS demonstrated a Spearman’s correlation coefficient of 0.92 with percent body fat as determined by DEXA, and a correlation coefficient of 0.74 with body weight (22). The strong correlation with both fat percentage and body weight observed in this principal study supports the high validity and reliability of BCS as a measure of feline adiposity and overall body condition (22).

In addition to its well-established utility in evaluating obesity, BCS along with muscle scoring is equally valuable for assessing conditions at the opposite end of the weight spectrum. In clinical practice, abnormal weight loss is often indicative of underlying pathological conditions such as cancer and hyperthyroidism (20, 22). Regular assessment of BCS along with other metrics facilitates early identification of these diseases by detecting subtle or significant reductions in body condition that may precede overt clinical signs. Moreover, in cases of suspected animal cruelty or neglect/inadequate care, BCS assessments provide crucial evidence. Veterinary practitioners and animal welfare officers utilize BCS as a tool to substantiate claims of inadequate nutrition or improper care, by objectively quantifying the degree of malnutrition or emaciation thereby informing legal proceedings, interventions, and decisions regarding animal custody and rehabilitation (30–34). The implementation of routine BCS evaluations significantly enhances the capacity of veterinary professionals to address both clinical and welfare concerns associated with abnormal body weight loss, supporting informed decision-making and promoting improved health outcomes and welfare standards for animals.

Despite the established utility of BCS in feline health management, BCS assessments have several limitations as they are a semi-quantitative and subjective system that depends on the training and experience of the evaluator. The inter-evaluator variability, which may compromise the consistency and reliability of BCS assessed by different clinicians at various levels of training, has not been comprehensively addressed in cats (35, 36). Digital imaging for BCS assessment holds significant promise not only for telemedicine applications, but also for enhancing the feasibility and robustness of large-scale research studies. Nevertheless, the reliability of assessments based solely on images requires rigorous evaluations to ensure accuracy, consistency, and clinical adaptability. Therefore, the goals of this study are to determine whether BCS can be accurately assessed solely from photographs of cats, and to measure inter-evaluator bias in visually assessed BCS.

## Materials and methods

### Animals, body weight measurement, and BCS judgment

Thirty-eight client-owned cats were enrolled with owner’s consent through Auburn University Veterinary Clinic (see Supplementary Data S1 for breed, age, sex, and spay/neuter status). Cats were recruited during routine wellness examinations, vaccinations, or nutritional consultations. All subjects were adults aged 6 months or older. Cats that had received antibiotics or been hospitalized within the previous 3 months were excluded from the study. BCS measurements were determined through clinical evaluations that included palpation. Evaluators were independently recruited for their experience in general practice and feline medicine and research or for their interest in feline medicine as professional students and included a total of 9 independent evaluators from Auburn University College of Veterinary Medicine, including 5 clinicians (2 community practice clinicians with over 5 years of experience, DVM, DABVP, 1 feline specialist, DVM, and 2 feline obesity research clinicians, DVM, PhD, DACVP) and 4 doctor of veterinary medicine (DVM) students (first- and second-year students recruited through the Student Feline Veterinary Medical Association club). These scores are designated as CA-BCS (Clinically Assessed Body Condition Scores). Prior to the beginning of this study, a training session (see training slides in Supplementary Data S2) was held for the 4 DVM students to familiarize them with the 9-point scale metric for cat BCS measurements (22). Body weight was also measured for these 38 cats at the time of BCS judgment (Supplementary Data S1). The study was approved by the Auburn University Institutional Animal Care and Use Committee (IACUC) with protocol number PRN 2022–4059.

### Study design of BCS assessment from visual cues only

Two sets of cat images, designated as BCS test sets (BTS)1 and BTS2, were compiled to create two BCS assessment quizzes for evaluating the inter-evaluator bias in BCS visual assessment. The same 9 BCS judges in this study were asked to assess the BCS of the BTS1 set (internet-sourced images) prior to the initiation of this study in April 2022. Criteria for internet sourced images included images of cats with clear and unobstructed views of the cat from the dorsal or lateral projections. Additionally, the judges also evaluated the BTS2 set February 2024, based solely on photographs of the 38 enrolled cats, approximately 2 years after the completion of the CA-BCS assessment. In BTS2 two images from each cat were used for visual assessment, including photographs taken directly above the cat (from the dorsal aspect) and from the side (lateral aspect). All images were taken either by the owner or the evaluator within 48 h of clinical assessment.

### BCS test set 1

A total of 46 cat photographs were collected from various online sources to create the BTS1 dataset for BCS evaluation (Supplementary Data S3). To ensure data integrity and validate scoring consistency, duplicate image pairs were created for 4 cats. These duplicates were generated by flipping or cropping the original images and altering the background color (see IDs in Supplementary Table S1). These modified images were randomly inserted into the stack, resulting in a total of 50 cats in the BTS1 collection (Supplementary Data S3). BTS1 was administered to 9 evaluators, who were asked to provide BCS judgments based on these images along with their confidence level (Supplementary Data S4, S5). Confidence levels were provided by each evaluator to gage their level of certainty of their own assessment for each cat. A confidence level A was selected if the evaluators are 100% confident in their single BCS score (recorded as 5 = 5 in the raw data). Level B was used for cases where two adjacent scores were considered equally likely (recorded as 5 = 6) for the evaluator. If the evaluator was unsure about two adjacent scores but leaned toward one, Level C is recorded (noted as 5 > 6). The keys (putative correct judgments) were determined based on the BCS judgments from Scorers A and B, both of whom are senior clinicians. Senior clinicians are defined as clinicians with at least 5 years of experience specializing in feline practice and feline research. These BCS measurements, determined solely through visual assessment, are defined as VA-BCS (Visual Assessment-based Body Condition Scores).

### BCS test set 2

BTS2 contains photographs of 38 client-owned cats (obtained with consent), which were taken from both the top and side views during routine wellness visits at Auburn University veterinary clinic or provided directly by the owners. Two photographs were duplicated and altered to ensure data integrity and validate scoring consistency (image pairs 4 ~ 33 and 11 ~ 23, see Supplementary Data S1), and the final BTS2 set consists of photographs of 40 cats. CA-BCS of these 38 cats was already determined by one of the 9 evaluators. Approximately 1 year after the study concluded, the same 9 evaluators assessed the BTS2 set solely based on the photographs without any additional information to obtain 9 VA-BCS per cat (Supplementary Data S6). This allowed for comparisons of assessments using clinical assessment and visual assessment only and then reviewing the visual assessment scores after an extensive period of time and in a new arrangement. Of these, the VA-BCS determined by the original clinical scorer, who judged CA-BCS a year earlier, was defined as VA-BCS_OCS (Visual Assessment-based BCS from the Original Clinical Scorer). VA-BCS_OCS represents a reassessment of the CA-BCS by the same scorer after a one-year interval, based solely on photographs.

### Obesity diagnosis for client-owned cats

The CA-BCS assessments of 38 client-owned cats ranged from 4 to 9. Scores of 4 ~ 5 were classified as normal, representing ideal body weight (IW). Scores of 6 ~ 7 were diagnosed as overweight (OW) but not obese, while scores of 8 ~ 9 were diagnosed as obese (OB) (20, 22, 23).

### Statistical analysis

**Figure 1:**
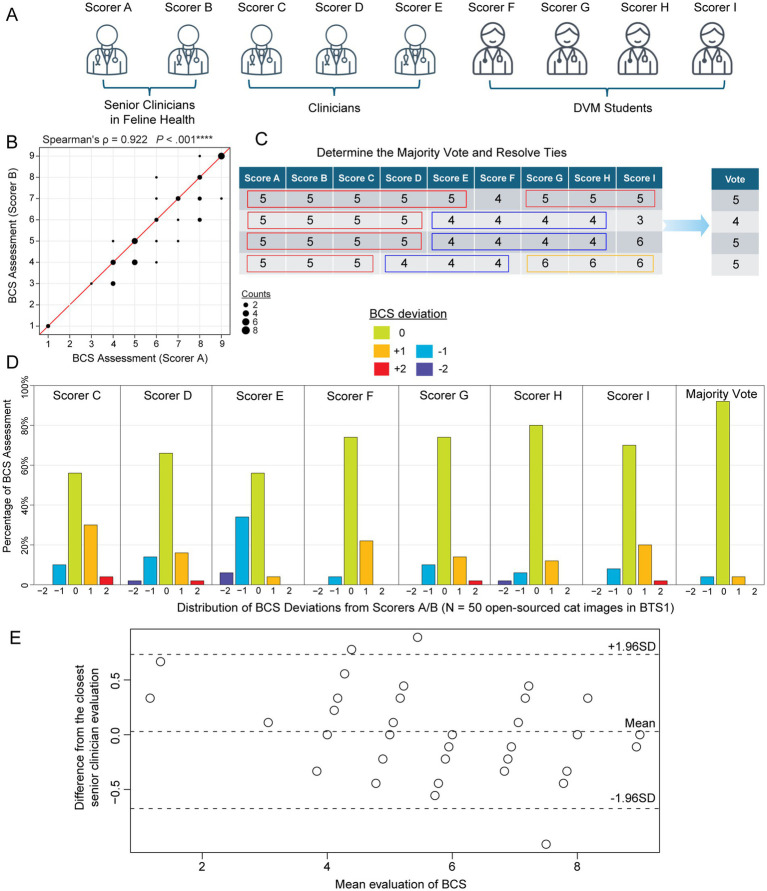
Feline body condition score (BCS) assessments of 50 internet-sourced cat images in BTS1 by 9 evaluators. **(A)** Nine evaluators grouped by their clinical experience: senior clinicians in feline health (Scorers A and B), Clinicians (Scorers C, D, and E), and DVM students (Scorers F, G, H, and I). **(B)** Scatterplot illustrates the correlation between the BCS assessments of Scorer A and Scorer B for the cat images in BTS1 cohort (BCS Test Set). The strong positive correlation (Spearman’s correlation coefficient *ρ* = 0.922, *p*-value < 0.00001) suggests high consistency between the assessments of these two senior clinicians in feline health. **(C)** Schematic table demonstrating the majority vote score metric, which is defined as the most common score among evaluators. In cases where there is a tie, the remaining scores are used to resolve it and determine the final score. **(D)** Barplots depict the distribution of deviations in BCS assessments compared to the judgments by Scorers A and B, which serve as the answer key in judging other scorers/ evaluations. Each subpanel represents a different scorer (C, D, E, F, G, H, and I) to show the percentage of assessments that deviated from Scorers A/B by −2, −1, 0, 1, or 2 points. **(E)** Bland–Altman plot illustrating the agreement between the average visually assessed BCS scores from the seven evaluators (Scorers C-I) and the closest reference BCS (Scorer A or B) across 50 internet-sourced cat images. The solid horizontal line indicates the mean difference between the evaluator and reference BCS, reflecting minimal systematic bias. The dashed lines represent the limits of agreement (±1.96 standard deviations), highlighting the range within which most differences fall.

Mean deviation of VA-BCS from the key assessment and the standard error were performed using the statistical software package R, version 3.6.3 (37). Spearman’s rank-order correlation coefficients between body weight and BCS assessment across different scorers and approaches, and inter-assessment correlations, as well as their *p* values were calculated using the cor.test function in the R programming environment. To compare the differences between repeated BCS assessments, the non-parametric Wilcoxon signed-rank tests were performed using the wilcox.test function in R (38, 39). Mann–Whitney U test was used to compare two groups of BCS deviations between BTS1 and BTS2 datasets (40). For VA-BCS assessed by nine scorers, a majority vote (VA-BCS_MA) scored was generated as another metric for final decision-making, which was determined by the most frequent value of BCS among the nine VA-BCS scores (Figure 1). Concordance of VA-BCS assessment between scorers was estimated by Kendall’s coefficient of concordance W using the function kendall in R package irr (41). To compare two dependent Spearman’s correlation coefficients, the R function twoDcorR was used with 5,000 bootstrap (42). Plots in all figures were generated using the ggplot2 package in R (43).

## Results

### BTS1 demonstrates low inter-evaluator bias of visually assessed BCS using internet-sourced cat images

For the internet-sources cat images in BTS1 (see Materials and Methods; Supplementary Data S3), a Spearman’s correlation of 0.922 (*p* < 0.001) between senior clinicians’ (clinicians specialized in feline practice and research; see Materials and Methods) assessments (Figures 1A,B), was observed indicating a high level of agreement and reliability. Thus, senior clinician judgments (BCS range 1 ~ 9 with a median of 6) were used as the answer key to evaluate the other seven scorers’ performance and consistency. Additionally, the majority vote was defined as a statistical measure of BCS among this group of 9 evaluators, by identifying the most frequent BCS assessment (Figure 1C and Supplementary Data S5). Variability in VA-BCS assessments was observed among different scorers (Figure 1D). However, it is noteworthy that more than 50% of the evaluations showed no deviation from the scores provided by the senior clinicians (scorers A/B). Among all VA-BCS evaluations, 29% demonstrated an absolute deviation of 1 point, 2.9% exhibited an absolute deviation of 2 points, and no assessments exhibited deviations of 3 or more points (Figure 1D). Three clinicians (Scores C, D, E) had a mean deviation of 0.45 with a standard error of the mean (SEM) of 0.08, whereas the four DVM students had a mean ± SEM of 0.28 ± 0.07 (Table 1). To evaluate inter-evaluator concordance, Kendall’s coefficient of concordance (W) was calculated for the VA-BCS scoring matrix and yielded a value of 0.903 (*p* < 0.001), indicating a high degree of agreement among the 9 evaluators as visualized in the Bland–Altman plot (Figure 1E). This low level of among-scorer variability indicated that all evaluators were able to assess BCS in a consistent manner, similar to the experienced senior clinicians. Notably, the majority vote can further enhance the consistency of BCS assessment, with more than 90% of the vote agreed perfectly with senior clinicians’ judgment (Figure 1D). The majority vote score had the best performance with a mean ± SEM of 0.08 ± 0.04 (*p* < 0.05, compared to all other Scorers, Wilcoxon signed-rank test; Table 1).

**Table 1:** Performance of BCS visual assessment in terms of deviation from senior clinical evaluations (BTS1) and clinical assessment of BCS for nine evaluators.

	BTS1		BTS2	
Scorer	Deviation (mean ± SE)	*P-*value (compared to vote)	Deviation (mean ± SE)	*P-*value (compared to clinical scorer’s deviation)*
Scorer A	n/a	n/a	0.47 ± 0.10	0.5577
Scorer B	n/a	n/a	0.50 ± 0.10	0.4662
Scorer C	0.48 ± 0.08	0.00002	0.61 ± 0.13	0.2405
Scorer D	0.38 ± 0.08	0.00068	0.82 ± 0.15	0.0237
Scorer E	0.50 ± 0.06	0.00002	0.66 ± 0.12	0.0995
Scorer F	0.26 ± 0.07	0.00869	0.53 ± 0.12	0.4992
Scorer G	0.28 ± 0.07	0.00825	0.61 ± 0.12	0.1709
Scorer H	0.22 ± 0.07	0.04125	0.68 ± 0.13	0.0989
Scorer I	0.32 ± 0.07	0.00254	0.63 ± 0.10	0.0768
Vote	0.08 ± 0.04	n/a	0.42 ± 0.09	0.7212

*Deviation (mean ± SE 0.39 ± 0.10; *N* = 38 client-owned cats) between clinical scorers’ evaluation from visual assessment in 2024 and their evaluation from palpation in 2022.

### Validation of low BCS assessment bias using photographs of client-owned cats

**Figure 2:**
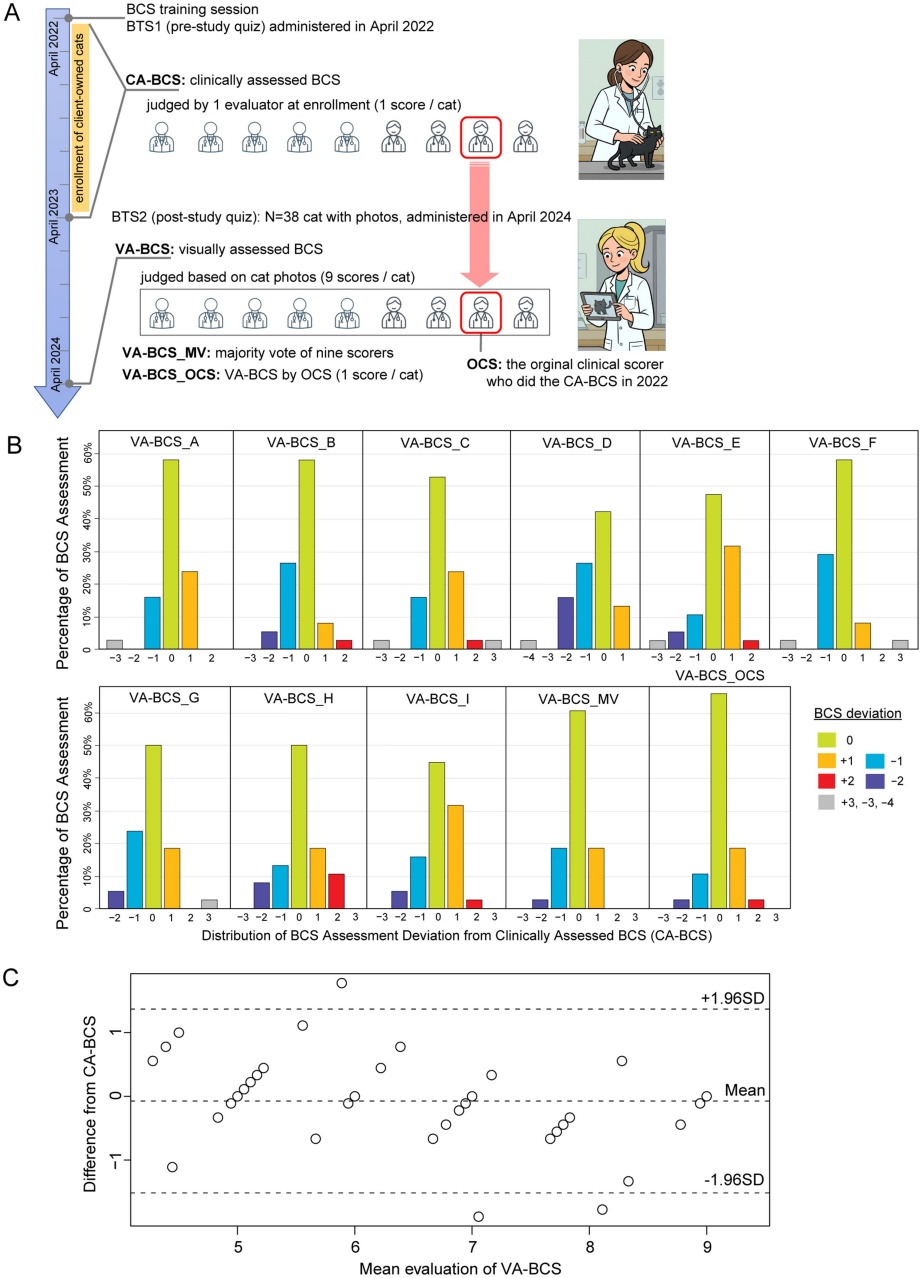
Clinically assessed BCS (CA-BCS) through palpation of 38 client-owned cats compared to visual assessments (VA-BCS) by 9 evaluators. **(A)** Schematic illustrations of the study design. BTS1 (BCS test set 1) images were administered to 9 evaluators shortly after the training session in April 2022. Client-owned cats were enrolled in this study between April 2022 and April 2023. At enrollment, CA-BCS was assessed through palpation in clinical settings by 1 scorer, who was known as the Original Clinical Scorer (OCS). After the conclusion of the enrollment period, BTS2 dataset consisting photographs of 38 client-owned cats from top and side view angles were compiled. BTS2 was administered to the same 9 evaluators to collect VA-BCS data (9 scorers per cat) in April 2024, 2 years after BTS1 assessment. VA-BCS_MV refers to the majority vote among the 9 scores. VA-BCS_OCS refers to the VA-BCS assigned by the original clinical scorer (OCS) who previously determined the CA-BCS during the clinical evaluation. **(B)** Barplots illustrate the distribution of deviations between CA-BCS and VA-BCS evaluated by each scorer (A-I), the majority vote of the 9 scorers (VA-BCS_MA), and the VA-BCS determined by the original clinical scorer (VA-BCS_OCS). The *x*-axis represents deviation categories ranging from −4 to +3, while the *y*-axis denotes the percentage of VA-BCS assessments within each category. **(C)** Bland–Altman plot comparing the mean VA-BCS for 9 evaluators with CA-BCS for 38 cats. The solid line represents the mean difference between VA-BCS and CA-BCS, indicating minimal bias between the two assessments. The dashed lines represent the 95% limits of agreement (±1.96 SD).

CA-BCS in the BTS2 dataset (Figure 2A) has a range from 4 to 9 with a median score of 6. To determine whether the low inter-evaluator bias persisted in VA-BCS assessments of client-owned cats, Kendall’s W was calculated and found to be 0.788 (*p* < 0.001), indicating good level of concordance among the 9 scorers (Figures 2B,C). Reliability of their scoring was assessed by deviation of the VA-BCS from the CA-BCS. The mean ± SEM of deviation was 0.61 ± 0.04 for 9 scorers combined, which was higher than the deviation observed in BTS1 by 0.09 (*p* < 0.001, Mann–Whitney U test). The VA-BCS values assessed by the original clinical scorer (VA-BCS_OCS) had the smallest deviation to CA-BCS, with a mean ± SEM of 0.39 ± 0.10 (Figure 2B and Table 1), which was not significantly different from the BTS1 assessment (*p* = 0.72, Mann–Whitney U test). The majority vote (VA-BCS_MV) had the smallest mean deviation from CA-BCS (0.42) compared with all 9 individual scorers (Table 1), and VA-BCS_MV demonstrated comparable performance as VA-BCS_OCS (*p* = 0.78, Mann–Whitney U test; Figure 2).

### Both palpation-based and visual BCS assessments demonstrate strong correlations with body weight

**Figure 3:**
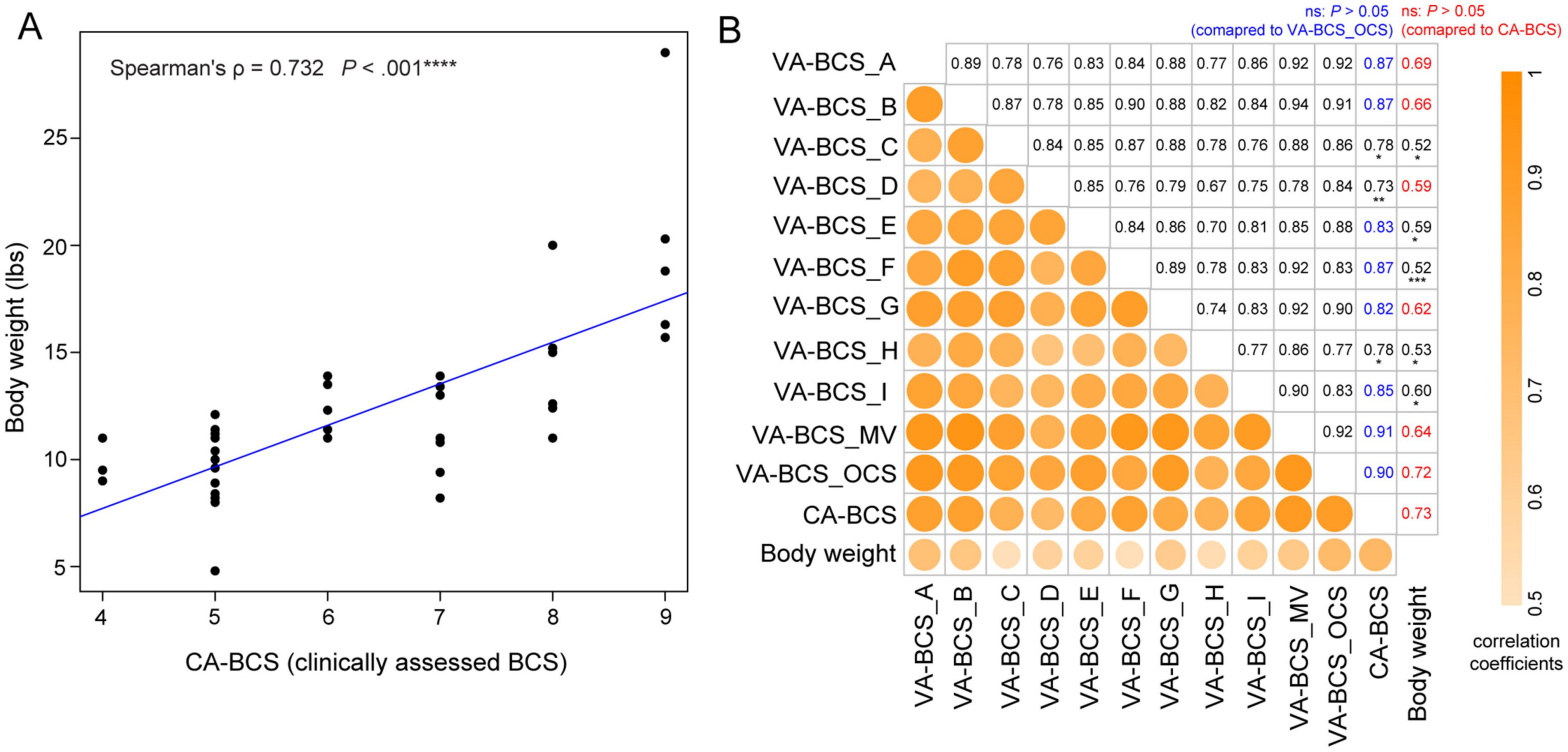
Correlation between visual assessments-based body condition scores (VA-BCS), clinically assessed BCS (CA-BCS), and body weight measurements in 38 client-owned cats. **(A)** Scatterplot illustrates the correlation between CA-BCS (*x*-axis) and body weight (*y*-axis). Spearman’s correlation coefficient ρ and *p* value were computed. A fitted linear regression line is plotted in blue. **(B)** Correlation matrix summarizing the relationships among BCS assessments and body weight across different scorers and scoring methodologies. Variables are arranged from top to bottom and left to right in the following order: VA-BCS from 9 individual scorers (Scorers A through I), the majority vote (VA-BCS_MV), the original clinical scorer’s VA-BCS assessment (VA-BCS_OCS), clinically assessed BCS (CA-BCS), and body weight. The lower-left triangle displays the strength of pairwise Spearman’s correlation coefficients using color intensity and circle size, whereas darker and larger circles indicate stronger correlations. The corresponding numerical values of these correlation coefficients are presented in the upper-right triangle. In the CA-BCS column, correlations of VA-BCS scores not significantly different from VA-BCS_OCS are shown in blue (*p* > 0.05, bootstrap test of two Spearman’s ρ, overlapping case). In the body weight column, correlations of VA-BCS scores not significantly different from CA-BCS are shown in red (*p* > 0.05). Statistically significant differences are indicated in black with asterisks denoting the level of significance: *p < 0.05; **p < 0.01; ***p < 0.001. This matrix highlights the robustness of visual BCS assessments across scorers and their strong concordance with both CA-BCS and body weight.

In the BTS2 dataset, body weight was measured concurrently with the CA-BCS evaluation (see Materials and Methods), enabling the investigation of the association between body weight and BCS. Despite the considerable variability in age, sex, and breed among the client-owned cats in the BTS2 dataset (Supplementary Data S1), their body weight exhibited a strong correlation with CA-BCS (Spearman’s *ρ* = 0.732, *p* < 0.001; Figure 3A), which is in remarkably close agreement with the correlation reported by Laflamme (Spearman’s ρ = 0.737). (22) This strong correlation with body weight justified the use of CA-BCS data in this study to serve as a benchmark for evaluating the performance of VA-BCS in BTS2 dataset.

VA-BCS assessments conducted by 9 scorers all demonstrate strong correlation with CA-BCS (all ρ > 0.73, *p* < 0.001; Figure 3B), which are consistent with the high level of concordance and low inter-evaluator bias observed (Figure 2C) These findings reinforce the validity and reliability of VA-BCS as a proxy for palpation-based assessment across different evaluators. The majority vote (VA-BCS_MV) has the strongest correlation with CA-BCS (*ρ* = 0.91, *p* < 0.001; Figure 3B), which was comparable magnitude to that of VA-BCS_OCS, which is assessed by the original clinical scorer (*p* = 0.81, bootstrap test of two Spearman’s *ρ*, overlapping case). VA-BCS assessments from Scorers A, B, E, F, G, and I demonstrate similarly high correlations with CA-BCS, ranging from 0.82 to 0.87, with no statistically significant differences from either VA-BCS_OCS or VA-BCS_MV (*p* > 0.05 for all comparisons; Figure 3B). These findings indicate that the majority vote, as well as most of the individual scorers, performed equivalently to the original clinical scorer in VA-BCS assessments. As expected, CA-BCS exhibits the highest correlation with body weight among all BCS metrics (Figure 3B). Consistently, VA-BCS assessments also demonstrate statistically significant positive correlation with body weight. Specifically, VA-BCS_OCS, the visual assessment performed by the original clinical scorer, shows a strong correlation with body weight (ρ = 0.72, *p* < 0.001; Figure 3B), comparable to that of CA-BCS (*p* = 0.81, bootstrap test of two Spearman’s ρ). VA-BCS scores derived from majority vote and each of the 9 individual scorers are also significantly correlated with body weight, with correlation coefficient ρ ranging from 0.52 and 0.69 (*p* < 0.001; Figure 3B). Among these, scorers A, B, D, and G demonstrate performance comparable to CA-BCS, with ρ of 0.69, 0.66, 0.59, and 0.62, respectively (*p* > 0.05, bootstrap test of two Spearman’s ρ). Notably, their performance is equivalent to the majority vote approach, with a correlation coefficient of 0.64.

Deviation in BCS from solely visual assessment does not alter the clinical diagnosis of obesity

**Figure 4:**
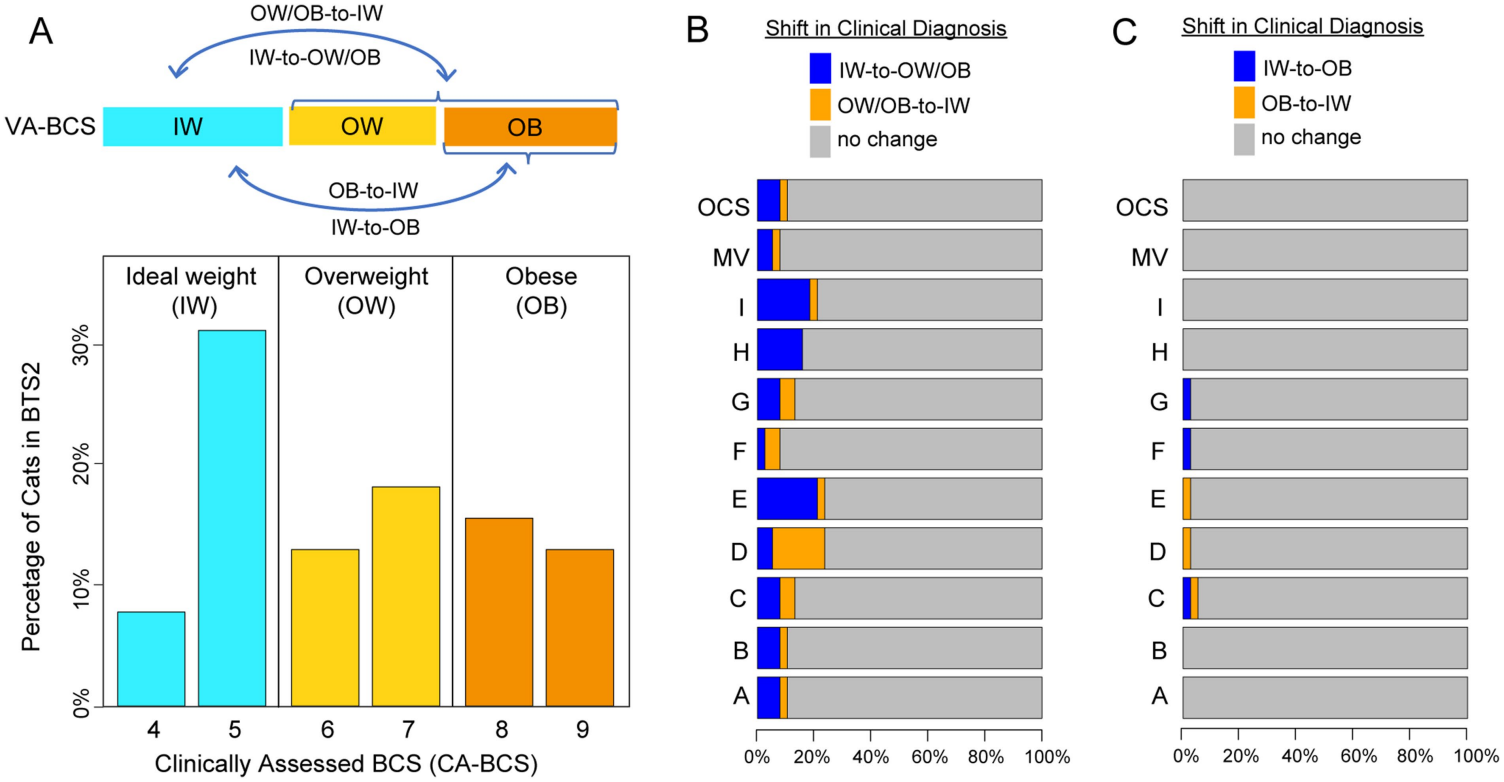
Clinical diagnosis shifts in body condition score (BCS) categories based on clinically assessed BCS (CA-BCS) and visual assessments-based BCS (VA-BCS). **(A)** Bar plot showing the distribution and diagnostic categories of CA-BCS for client-owned cats in the BTS2 dataset: Ideal Weight (IW, BCS 4 ~ 5, blue bars), Overweight (OW, BCS 6 ~ 7, yellow bars), and Obese (OB, BCS 8 ~ 9, orange bars). The percentage of cats (*y*-axis) is plotted for in each BCS category (*x*-axis). Arrows in the top panel illustrate potential misclassifications (subpanels B and C) between categories when evaluated through VA-BCS. **(B)** Horizontal bar plot showing the shifts in clinical diagnosis between CA-BCS and VA-BCS, highlighting transitions between IW and OW/OB categories. Bars represent the percentage of cats with no diagnostic change (gray) or shifts in diagnosis (blue: IW-to-OW/OB, orange: OW/OB-to-IW) for VA-BCS of each evaluator (A ~ I), the majority vote (MV), and the original clinical scorer (OCS). **(C)** Horizonal bar plot showing the shifts in clinical diagnosis between CA-BCS and VA-BCS, highlighting transitions between IW and OB categories. Bars represent the percentage of cats with no diagnostic change (gray) or shifts in diagnosis (blue: IW-to-OB, orange: OB-to-IW) for VA-BCS of each evaluator (A ~ I), the majority vote (MV), and the original clinical scorer (OCS).

Next, the reliability of VA-BCS in clinical diagnosis of overweight and obesity was assessed. Based on CA-BCS, 39.5% of the client-owned cats in BTS2 had ideal weight (IW, BCS 4 ~ 5; Figure 4A). Of the remaining cats, 28.9% were diagnosed as obese (OB, BCS 8 ~ 9), and 31.6% were diagnosed as overweight but not obese (OW, BCS 6 ~ 7). Across the 9 individual scorers, deviations in VA-BCS from CA-BCS resulted in an average misclassification rate of 15.5% between IW and OB/OW categories (Figure 4B), which was generally reliable but with a considerable margin of error. The majority vote had a misclassification rate of 7.9%, outperforming individual scorers; however, this improvement did not achieve statistical significance (*p* = 0.30, Fisher’s Exact Test). In feline obesity research, ideal weight (IW) is often compared with the obese (OB) group. Consequently, the misclassification rate between these two categories was evaluated. On average, the 9 individual scorers had a misclassification rate of only 1.75%, with no cats being misclassified between IW and OB categories by Scorer A, B, H, I, or majority vote (Figure 4C). This extremely low misclassification rate between IW and OB indicates a high level of accuracy and reliability in diagnosing feline obesity using VA-BCS.

### Impact of photograph angle on VA-BCS misclassification: examples from duplicated cat images

**Figure 5:**
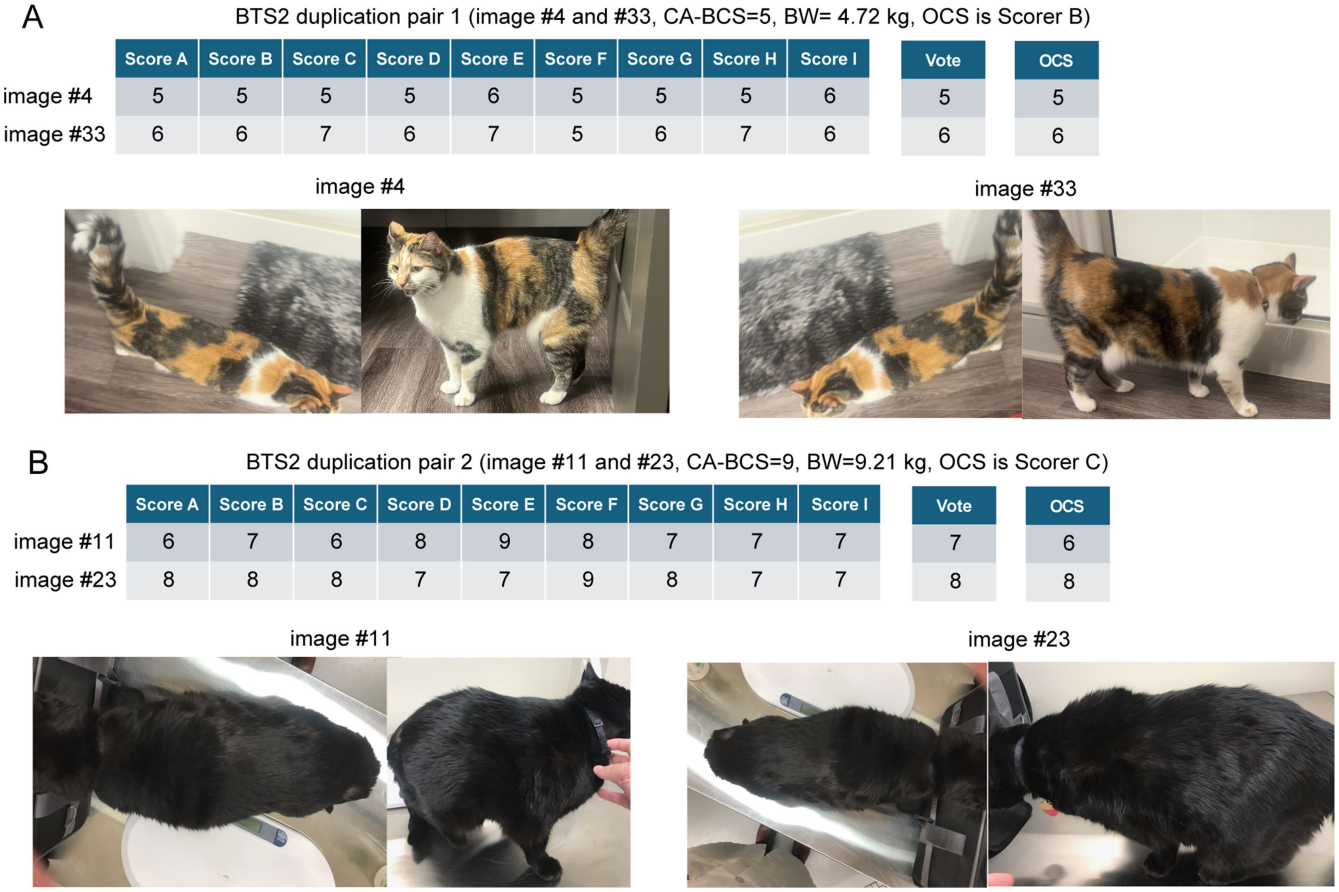
Variability of visual assessments-based body condition scores (VA-BCS) in duplicated image pairs from the BTS2 dataset. **(A)** VA-BCS and majority vote for duplicated pair 1 (Images #4 and #33) from a female calico cat with a clinically assessed BCS (CA-BCS) of 5 and a body weight (BW) of 4.72 kilograms (kg). Scorer B is the Original Clinical Scorer (OCS). Individual Scorers A ~ I performed VA-BCS assessments, as shown in the table. Image #4 received a majority vote of 5, consistent with the CA-BCS and VA-BCS_OCS. In contrast, Image #33 scored slightly higher, with a majority vote of 6 and a VA-BCS_OCS of 6. The side-view image for Image #33 highlights a broader body profile, which may account for the higher VA-BCS scores compared to Image #4. **(B)** VA-BCS and majority vote for duplicated pair 1 (Images #11 and #23) from a male black cat with a CA-BCS of 9 and a BW of 9.21 kg. Scorer C is the OCS. Individual Scorers A ~ I performed VA-BCS assessments, as shown in the table. Image #11 received a majority vote of 7 and a VA-BCS_OCS of 6, which was lower than the CA-BCS of 9, suggesting an underestimation. Conversely, Image #23 achieved both a majority vote and a VA-BCS_OCS of 8, closely aligning with the CA-BCS. The differences in scoring between these images may be attributed to variations in the side-view angles. Image #11 depicts the cat in a bent posture, which obscures the visibility of abdominal fat deposits, potentially leading to an underestimation of BCS. In contrast, Image #23 provides a clearer side profile that better highlights the characteristic “small head syndrome” commonly observed in obese cats, resulting in a more accurate assessment of BCS.

To evaluate whether the angle of photographs influences the visual assessment of BCS, two duplicated pairs with varying angles were incorporated into the BTS2 dataset (see Materials and Methods). The first pair comprises Image #4 and Image #33 of a female calico cat with a CA-BCS of 5 (Figure 5A). The majority votes, BCS score 5 for Image #4 and score 6 for Image #33, perfectly aligned with the VA-BCS_OCS assessments (Figure 5A). This suggests that while variations in photographs influenced VA-BCS judgments, the overall impact remained minimal (less than 1) and consistent across different scorers (Figure 5A).

In contrast, a male black cat exhibited the largest deviation between CA-BCS and VA-BCS_OCS among all 38 client-owned cats, with a CA-BCS of 9 and body weight of 9.21 kg (Figure 5B). Two sets of photographs of this cat, Image #11 and Image #23, were included in the BTS2 dataset. Image #11 received a majority vote of 7 and a VA-BCS_OCS of 6, which was lower than the CA-BCS of 9, suggesting an underestimation. Conversely, Image #23 achieved both a majority vote and a VA-BCS_OCS of 8, closely aligning with the CA-BCS. The differences in scoring between these images may be attributed to variations in the side-view angles. Image #11 depicts the cat in a bent posture, which obscures the visibility of abdominal fat deposits, potentially leading to an underestimation of BCS. In contrast, Image #23 provides a clearer side profile that better highlights the characteristic “small head syndrome” commonly observed in obese cats, resulting in a more accurate assessment of BCS. Image #23 was assigned a BCS of 8 by both the majority vote and the VA-BCS_OCS assessments (Figure 5B), demonstrating perfect consistency and no misclassification in the diagnosis (as an OB cat). Cat breed, age and coat color varied in images of cats in BTS 1 and 2. Most of the cats in both data sets were short hair cats with one image of a long-haired cat included in BTS1 and two long-haired cats included in BTS2. There were too few cats with long hair coats to evaluate the effect of hair coat length on visual assessment. Our data provides some insight into the impact on coat length and color on visual assessment of BCS; however future studies are needed to better address these questions.

## Discussion

### BCS based on visual assessments alone: reliability, robustness, and limitations

BCS assessed by veterinarians in a clinical setting demonstrates a stronger correlation with percent body fat mass than with actual body weight (20), making BCS important in routine evaluation of health in particularly susceptible populations such as domestic cats. In this study, we aimed to assess the extent to which visual cues contribute to BCS judgments, as reflected by the reliability of VA-BCS assessed from cat images compared to CA-BCS evaluations conducted through actual palpation. We identified strong correlations between VA-BCS and CA-BCS across client-owned cats, suggesting that VA-BCS is a robust and reliable proxy for accurately estimating true CA-BCS. Besides, high degree of concordance of VA-BCS among scorers further supports the broader adoption of the VA-BCS method in practice. In terms of correlation with actual body weight, CA-BCS demonstrated the strongest association, reinforcing the utility of palpation-based assessments in accurately reflecting an animal’s physical condition. VA-BCS_OCS, the visually assessed BCS assigned by the same evaluator who previously determined the CA-BCS in a clinical setting, demonstrated the second-highest correlation with body weight. This result is expected, as VA-BCS_OCS evaluators had prior direct interaction with the cats, potentially incorporating additional contextual information into their assessments. Notably, VA-BCS assessments from two-thirds of the evaluators (three clinicians and three DVM students) exhibited correlation strengths comparable to VA-BCS_OCS, indicating that their visual assessments performed as accurately as those of the original clinical scorer. Furthermore, nearly 50% of evaluators (three clinicians and one DVM student) demonstrated VA-BCS correlations with body weight that were statistically similar to CA-BCS, suggesting that trained professionals can achieve reliable BCS evaluations through visual assessments alone. In this study, none of the cats had a BCS less than 4 which means we were not able to fully assess the process in cats that are underweight. An additional limitation of this study is the use of body weight as the main parameter for body composition instead of fat mass in clinically assessed cats. Overall, the strong agreement between the two methods of BCS assessment supports the feasibility and validity of VA-BCS as a practical alternative to palpation-based BCS, particularly in cats that are obese.

Previous studies have attempted to estimate body composition from photographs in dogs, and similar to the findings in this study showed that in experienced observers, there is moderate to good correlation between the visual BCS and body composition (44). The canine study also demonstrated that factors such as age, sex, breed, coat length and coat color did not significantly affect the visually assess BCS. Interestingly this study also included 5 non-veterinary trained observers highlighting the importance of training for veterinary health professionals (44). Ultimately, our study is the first to evaluate the use of VA-BCS as an effective method for assessing BCS in domestic cats, and our findings support its potential applications in both clinical practice and research settings.

### Inter-evaluator bias in BCS scoring may influence the outcomes of feline obesity research, contingent upon the specific experimental design

In this study, we identified significant inter-evaluator bias in VA-BCS assessments, with average deviations ranging from 0.52 to 0.83. These findings are similar to what is reported in interobserver variability in other species. Notably, the observed level of bias in this study is lower than initially anticipated, with a nominal average deviation of less than 1 point and a statistically significant deviation below 2 points. Therefore, we recommend a minimum BCS difference of 2 points when comparing groups in feline obesity studies to ensure reliable and meaningful distinctions. Furthermore, inter-evaluator bias influenced diagnostic categorization in feline clinical practice, resulting in a 15% misclassification rate between the IW category (BCS 4 ~ 5) and the OW/OB category (BCS ≥ 6). However, when distinguishing between ideal weight and obesity (BCS 8 ~ 9), misclassification occurred in fewer than 2% of cases, indicating that inter-evaluator bias has a minimal impact on the diagnosis of obesity, even when VA-BCS assessments are conducted by multiple veterinary professionals. These findings align with what has been previously reported in other species (13, 44) and affirms the utility of VA-BCS as a trustworthy diagnostic approach for feline health, capable of supporting accurate clinical decision-making and robust research endeavors.

### Reduction of inter-evaluator variability of VA-BCS: training and standardization procedures

In research and clinical practice, several approaches can be applied to reduce the inter-evaluator bias. First of all, the development and implementation of comprehensive training sessions ensure that all evaluators attain a uniform understanding of the feline BCS system. In this study, DVM students participated in a one-hour training session and performed equally well to clinicians. Previous studies reported greater interobserver bias, but also included observers that lacked any professional veterinary training, underscoring the importance of adequate training of students in BCS assessments (44). A set of training slides specifically designed for the 9-point scale BCS evaluation is included in Supplementary Data S2. Second, internet-sourced cat images (BTS1) can be utilized as a calibration dataset to identify evaluators’ tendencies to overestimate or underestimate VA-BCS. Evaluators who consistently produce outliers can be identified and retrained to enhance the overall accuracy and reliability of the VA-BCS assessments. Third, we believe that high-quality photographs captured under standardized conditions could significantly enhance the consistency of VA-BCS evaluations. In this study, we demonstrated that the angle of the photograph adversely affected the VA-BCS judgment for a male black cat, leading to potential misclassification. While coat color and length were not reported to be impactful in previous studies in dogs (44), we feel these variables and additional factors including image quality may also impact visual BCS classification in cats. Therefore, implementing a uniform imaging protocol, including multiple consistent angles, such as top, front and side views, as well as standardized lighting and image resolution, along with appropriate observer training, will minimize variability in VA-BCS assessments, thereby improving the reliability and accuracy of body condition evaluations across different evaluators and settings. Finally, we demonstrated that the majority vote method achieved the highest performance by significantly reducing the deviation of VA-BCS from CA-BCS. We would recommend that a committee assessment with majority vote be used when making important research and clinical decisions that rely on BCS assessment. As a score aggregation approach, this consensus-based method effectively mitigates individual bias to achieve more reliable BCS assessments. Additionally, cat images can be collected and stored in the medical record, allowing multiple veterinary professionals to evaluate the same set of images and determine the majority vote as the final assessment as well as compare changes in individual cats over time. Digital image analysis has been successfully implemented in ruminant medicine with methods to reduce interobserver bias in BCS assessment (13, 14, 45–47), providing groundwork for the use of automated assessment in companion animals.

### Potential clinical applications of VA-BCS

The current clinical standard for BCS evaluation, CA-BCS, remains the most reliable method for semi-quantifying the body conditions in cats. This study compared the most clinically feasible tool to assess body conditions (CA-BCS) with a method (VA-BCS) that has value in BCS assessment when palpation is not feasible, allowing greater flexibility for the patients, clients, and clinicians. Cats are notoriously challenging to position and handle in clinical settings without specifically trained and experienced personnel. While acquiring high quality photographs can be a limiting factor for some owners, regulations for certain quality photos are likely important for future applications. In this study we did not provide detailed instruction for owners or clinicians on the image acquisition process. We only requested photos from the dorsal and lateral views. This created some limitations with certain photos. Regardless, there was still good correlation with the physical assessment suggesting that image quality may not be as significant a factor. Defining best practices for photograph angles, lighting, and resolution may be essential for ensuring consistency and clinical applicability across diverse veterinary settings with further studies needed to evaluate how these factors may affect visual BCS assessments, overall. These findings demonstrate that VA-BCS serves as a highly accurate and reliable alternative, with an average deviation from CA-BCS of less than one point. VA-BCS are particularly useful in the following scenarios: (1) telemedicine applications: in remote consultations where physical palpation is not feasible, VA-BCS provides a practical alternative for assessing body condition; (2) long-term health monitoring: when feline patients were examined by clinicians over time, photographic record based VA-BCS could be used as a tool to reduce individual bias and temporal bias and improve consistency in BCS evaluations; (3) consensus-based assessment: VA-BCS enables multiple clinicians to review the same set of images, facilitating majority vote methods that enhance reliability and minimize variability. Despite these advantages, further research is required to establish a standardized imaging protocol that optimizes the accuracy of visual BCS assessments.

### Future directions: machine learning approaches for automated image analysis in VA-BCS assessment

Our research has demonstrated the accuracy of VA-BCS assessments from veterinarians, providing a benchmark for integrating artificial intelligence technologies, such as machine learning (ML) algorithms, for automated image analysis systems to infer BCS. We intend to establish a comprehensive database of client-owned cat images, sourcing data both locally from our small animal teaching hospital and through images collected via a mobile application. This mobile application will deliver a computational inference of BCS to users/submitters, utilizing ML models. Such ML models will be developed to account for breed-specific morphological variations, anatomical landmarks in differentiate BCS categories, photograph angles and lighting conditions, to improve the accuracy of BCS assessments. A proportion of the dataset will include CA-BCS measurements, and we will have local veterinarians perform VA-BCS assessments on selected cat images to enhance the accuracy and efficacy of our ML model. Continuous validation refinement of the ML model through iterative training and cross-validation with CA-BCS data will ensure its robustness and generalizability across diverse feline populations, ultimately achieving comparable performance to senior clinicians in assessing cat BCS. The ML model will become an objective and reliable tool for BCS inference, facilitating remote veterinary consultations in telemedicine without the need for physical palpation. It can be integrated into pet video camera systems for continuous BCS monitoring, thereby enabling the systematic tracking of feline health conditions. Additionally, the ML approaches can support large-scale epidemiological studies through the rapid analysis of vast numbers of images, achieving scalability of research efforts aimed at understanding feline obesity trends and associated risk factors.

## Data Availability

The original contributions presented in the study are publicly available. Supplemental Data S1–S6 are available in the open-access AUrora repository (https://doi.org/10.35099/n3w5-p894).
